# Health literacy as mediator between perception of illness and self-medication behaviour among outpatients in the Kingdom of Saudi Arabia: implication to primary healthcare nursing

**DOI:** 10.1186/s12912-024-01950-9

**Published:** 2024-04-25

**Authors:** Eddieson Pasay-an, Reynita Saguban, Dolores Cabansag, Sameer Alkubati

**Affiliations:** 1 Maternal and Child Nursing Department, College of Nursing, University of Hail, Hail City, Hail Saudi Arabia; 2 Department of Mental Health Nursing, AJA campus, College of Nursing, University of Hail, Hail City, Saudi Arabia; 3 Medical-Surgical Nursing Department, College of Nursing, University of Hail, Hail City, Saudi Arabia

**Keywords:** Health literacy, Mediator, Perception of illness, Self-medication behaviour, Outpatients, Saudi Arabia

## Abstract

**Introduction:**

Perception of illness (PI) and self-medication (SM) have been thoroughly explored in the existing literature. However, there is a lack of understanding about the mediating effect of health literacy on PI and SM in a non-homogenous population like Saudi Arabia. As such, primary healthcare nurses who have constant interaction with the outpatients have difficulty addressing self-medication. This study aimed to investigate health literacy as mediator between PI and SM among outpatients in the Kingdom of Saudi Arabia (KSA).

**Methods:**

This study employed a cross-sectional approach and was conducted at 10 major primary healthcare (PHC) clinics serving 30 million individuals in 13 different regions of KSA. The 424 outpatients who participated in this study were selected through convenience sampling. Data collection started in November 2022 and concluded in February 2023.

**Results:**

The mean of the Brief Health Literacy Screening Tool, self-medication scale (SMS), and PI scores were 13.01 ± 3.32, 27.46 ± 7.01, and 45.56 ± 7.69, respectively. There was a significant relationship between the age and BRIEF scores (*p* = 0.039), and the level of education was significantly related to all variables, as were nationality and BRIEF scores (*p* = 0.001). Finally, occupation was significantly related to BRIEF and SMS scores (*p* = 0.001 and 0.003, respectively). Completing college and being non-Saudi had positively significant effects on health literacy (*p* < 0.01). The structural equation model (SEM) found no effect of PI on health literacy or SM behaviour (*p* = 0.263 and 0.84, respectively), but health literacy did have an effect on SM behaviour (*p*<0.001).

**Conclusion:**

Health literacy is an important factor in self-medication behavior and that PI is not directly related to health literacy or self-medication behavior, but that health literacy does influence self-medication behavior. Therefore, primary healthcare givers should promote public health literacy alongside the control of other conditions as one of the most effective ways to decrease the prevalence of self-medication and the risks associated with it.

## Introduction

The ability to collect, read, grasp, and apply healthcare information is necessary for an individual to make informed health decisions and follow treatment recommendations [1, 2]. To handle common health issues successfully, a person needs a set of personal health literacy skills [3]. Lack of correct information affects health outcomes; thus, everyone should have the chance to use accurate information to enhance their health and wellness. According to Weiss [4], lack of health literacy influences health outcomes because it makes people less inclined to practise good self-care [4]. Hence, increasing health literacy benefits one’s ability to communicate, make better health decisions, follow treatment instructions, and maintain good health. Increased patient–provider satisfaction and decreased healthcare system costs should result from all these aspects [5]. Psychosocial characteristics, such as socioeconomic status and coping, may be greatly impacted by how well one understands health information [6]. Age is among the key determinants of poor health awareness, and socioeconomic status, ethnicity, intellect, and education have all been considered important factors in earlier studies [7]. Health service providers should be aware of and able to meet the needs of their population’s health literacy, given the demonstrated link between poorer health outcomes and lack of health literacy [8].

SM is defined as the use of drugs, herbs, or home remedies on an individual’s own initiative or the advice of a third party without consulting a physician [9]. Unquestionably, many laypeople frequently self-medicate as a form of self-care because they are unaware of the potential adverse effects of such medications [10]. Insufficient treatment compliance, patient discontent with the healthcare practitioner, the patient’s lack of awareness of both their health status and their medicine [11], and having an initial treatment plan for a similar condition that underrates the disease [12] can all add to the reasons for self-medicating. In addition, poor health decisions, risky behaviour, less self-control, and more significant hospitalisation have all been linked to low health literacy competence [13].

A patient’s perception of illness (PI) is based on their cognitive evaluation and comprehensive understanding of a medical condition and its potential implications [14]. Negative PI is linked to increased future disability, a slower recovery, and a delayed return to work regardless of the severity of the medical condition or the existence of litigation. A positive PI has been linked to psychological wellbeing, a decreased need for benefits, and an earlier return to work [15, 16]. A person’s view and conceptualisation of having a condition constitute PI [17]. This perception may include both optimistic and pessimistic health perspectives that could influence how well someone can manage their illness and whether they believe it to be dangerous or under control [18]. In previous studies of physical illnesses, PI has been found to predict significant outcomes, such as time spent recovering and adherence to treatment [16].

Earlier studies have found a link between SM and health literacy. Kamram and associates [19], for instance, found that health status and knowledge of health were significantly correlated with SM. A link was also found between patient literacy and some medication-related behaviours and outcomes [20, 21]. Correspondingly, healthcare disparities are significantly influenced by low health literacy and thus impact almost every element of health [22]. Adult literacy is linked to inappropriate medicine usage, including nonadherence, overdosing, and misreading drug labels and instructions [23]. At the same time, Kim and colleagues’ findings showed that, in some self-management activities, patients with adequate health literacy underperformed in comparison to those with inadequate literacy [24]. Although PI and SM have been thoroughly explored in the existing literature, the mediating effect of health literacy on both in a non-homogenous population has not been fully explored.

Primary healthcare (PHC) seeks to reduce hospital visits [25], and practitioners have been successful in reaching their targets because many patients favour PHC. These facilities provide preventative and primary care for numerous medical disorders [26]. In context, PHC caregivers have played an important role in the prevention of disease and promotion of health by interacting with the general population. Further, even though PHC personnel have most contact with outpatients in the PHC unit, the extent of their role in addressing the SM problem has not been fully understood. Therefore, this study is crucial because it can offer PHC personnel a useful guideline for educational initiatives to increase public understanding of illness and the effects of SM.

In context, given the increased incidence of SM in the general population, the need to identify factors influencing behaviour change is an important part of practising healthy behaviour. Making reports available to policymakers and primary healthcare nurses to promote SM knowledge and literacy among the general adult population is consequently very important. This study aimed to investigate health literacy as a mediator between PI and SM among outpatients in the Kingdom of Saudi Arabia (KSA). It hypothesises that PI will have a positive association with SM and health literacy and that health literacy will have a positive association with SM behaviour. Lastly, PI affects SM beliefs and behaviours mainly through its influence on health literacy.

## Methods

### Design

This study employed a cross-sectional approach to investigate health literacy as a mediator between PI and SM among outpatients in KSA.

### Setting/sampling

The 10 major primary healthcare clinics that took part in the study service 30 million individuals in 13 different regions. These centres were selected using the guiding principle of including two major regions (Northern and Eastern), two medium regions (Hail and Aseer), and two small regions (Aljouf and Al-Ahsa), which, in terms of population density, are large, medium, and small, respectively. We selected these 10 primary healthcare centers with the following considerations: (1) the size and locations, which represent the large number of residents; (2) clinics with equal geographic distribution and high patient satisfaction; and (3) clinics with multicultural demographic representation, which includes patients from various backgrounds, which adds diversity and inclusivity to the samples. To estimate the prevalence of health literacy with a precision of 5% and a 95% confidence level, and we assume that the prevalence of health literacy in the population is 50%, this results 385 required sample size. The sample size was calculated based on the number of population (e.g. Northern region at 10 million population with 76 participants; Eastern region at 8 million population with 60 participants ) and convenience sampling was utilised. Because individuals were included through convenience rather than random sampling, the sample size was then expanded by 10% to accommodate contingencies such as non-response and/or anticipated dropouts, bringing the total sample size to 424 participants.

The participants of this study were outpatients who were visiting PHC centres for a check-up. The eligibility criteria were: (1) being at least 18 years old, (b) being able to read and comprehend English, (c) not being in any medical distress during the check-up, and (d) willingness to participate.

### Data collection

Data collection started after ethical clearance was received. The informed consent procedure was explained to the participants, as was the aim of the study, the extent of their participation, and their rights as participants. The participants were given a paper-based questionnaire during their visits to the PHC and asked to read the informed consent before answering it. A minimum of 15 min was given for participants to answer the questionnaire; they could extend that time if required. Data collection started in November 2022 and concluded in February 2023.

### Instruments

In addition to collecting the demographic characteristics of the participants, this study administered three questionnaires, as described below.

The first instrument is the self-rated Illness Perception Questionnaire (Brief IPQ) [14] was used to evaluate patients’ cognitive representations of their illness. The nine items on the Brief IPQ address consequences, timeline, personal control over treatment, identity over sickness, coherence, and emotional representation. The ninth question asks outpatients to list three things they believe to be the cause of their medical condition and is thus a causal item. However, in the current analysis this item was not utilised. Each item was scored from 0 to 10. An example of items in the questionnaire is ‘How much does your sickness affect your life?’ (Item 1), where 0 indicates ‘not at all’, and 10 implies ‘it has a significant impact on my life’. With a Cronbach’s alpha of 0.79, the sample’s reliability was deemed adequate. In the context of outpatient care, we used the condensed form of the IPQ to reduce questionnaire load on patients compared to the original IPQ which measures comprehensive perception of illness.

The second instrument is the BRIEF: Health Literacy Screening Tool, a validated four-item screening questionnaire developed by Haun et al. [27], evaluates the patient’s capacity to understand health information. The questions are: ‘How often do you get someone to study hospital documents with you?’; 2. ‘How comfortable are you completing medical forms on your own?’; 3. ‘How frequently do you find it difficult to grasp written information regarding your medical condition?’; and 4. ‘How confident are you filling out medical forms by yourself?’ Participants responded to each question on a 5-point scale. The BRIEF scale runs from 4 to 20, and three criterion levels for BRIEF scores were followed: inadequate (scores of 4 to 12), marginal (scores of 13 to 16), and adequate (scores of 17 to 20).

The third questionnaire assessed the SM beliefs and behaviour of outpatients, resistance to SM, comfort with SM, and views about letting events play out naturally was adapted from James and French [22]. Its nine items were answered on a 5-point Likert scale where 1 = Rarely, 2 = Not that often, 3 = Sometimes, 4 = Often, and 5 = Very often. Negative statements, such as items 7 and 8, were reversed.

### Ethics approval and consent to participate

This research was conducted with the approval of the Institutional Review Board of the University of Hail (H-2022-022). The participants were assured that all data gathered would be treated with the utmost confidentiality. All methods were carried out in accordance with relevant guidelines and regulations. Moreover, informed consent was taken from participants to participate in the study.

### Statistical analysis

The collected data were analysed using SPSS version 26, and frequency and percentage were used to represent the descriptive variables. The mean score and Pearson correlation findings were determined. The survey items were then analysed based on their mean scores. The relationship between PI and SM was also examined using structural equation modelling (SEM) to determine whether health literacy was a mediator.

## Results

Table 1 illustrates the demographic and occupational characteristics of the general population (*N* = 424). The mean participant age was 37.29 with a standard deviation (SD) of 12.09. The majority (47.6) were single, followed by married and separated (41.5% and 10.8%, respectively). Regarding educational level, more than one third did not complete college (38.2%) while less than one third completed college (29.0%). Individuals of non-Saudi nationality formed more than half of participants (51.7%). Approximately two thirds (65.6%) were in blue-collar occupations.

**Table 1:** Demographic characteristics of participants (*N* = 424)

Characteristics		*n*	(%)
Age	Mean ± SD	37.29 ± 12.09	
Marital Status			
	Single	202	47.6
	Married	176	41.5
	Separated/Divorced	46	10.8
Educational Level			
	Completed college	123	29.0
	Did not complete college	162	38.2
	Completed secondary	99	23.3
	Elementary	40	9.4
Nationality			
	Saudi	205	48.3
	Non-Saudi	219	51.7
Occupation			
	Blue-collar occupation	278	65.6
	White-collar occupation	146	34.4

Table 2 illustrates the means and SD of the scores of the health literacy (BRIEF) scale, resistance to self-medication (SMS) scale, and PI scale. The mean BRIEF, SMS, and PI scores were 13.01 ± 3.32; 27.46 ± 7.01, and 45.56 ± 7.69, respectively.

**Table 2:** Descriptive results on BRIEF, SMS, and PI scores

Scale	Range	Mean ± SD
**BRIEF**	4–20	13.01 ± 3.32
**SMS**	10–44	27.46 ± 7.01
**PIS**	29–66	45.56 ± 7.69

Table 3 shows a significant relation between all the studied variables and the scores of the health literacy (BRIEF) scale, resistance to self-medication (SMS) scale, and PI scale. There was a significant relationship between age and BRIEF scores (*p* = 0.039), while there were no significant relationship between age and resistance to SMS and PI scores (*p* = 0.970 and 0.786, respectively). Marital status was not related to the scores of health literacy (BRIEF), resistance to SMS, or PI scores (*p* = 0.256, 0.078, and 0.817, respectively) while the level of education was significantly related to all variables (*p* = 0.001, 0.029, and 0.005). There was a significant relationship between nationality and BRIEF scores (*p* = 0.001). Finally, occupation was significantly related to the BRIEF and SMS scores (*p* = 0.001 and 0.003, respectively). There was no significant relationship between the SMS scores and items of nationality and source of information (*p*˃0.05).

**Table 3:** Relationship between participants’ characteristics and BRIEF, SMS, and PI scores

Factors	Group	*N*	BRIEF		SMS		PI	
			Mean ± SD	*p*-value	Mean ± SD	*p*-value	Mean ± SD	*p*-value
**Age**		424	12.97 ± 3.32	0.039	27.46 ± 7.01	0.970	45.56 ± 7.69	0.786
**Marital Status**								
	Single	202	13.25 ± 3.32	0.256	26.66 ± 6.34	0.078	45.52 ± 7.52	0.817
	Married	176	12.71 ± 3.50		28.25 ± 7.86		45.76 ± 8.12	
	Separated/Divorced	46	12.78 ± 2.46		27.95 ± 6.05		44.95 ± 6.77	
**Educational Level**								
	Completed college	123	14.27 ± 3.47	0.001	28.92 ± 7.12	0.029	45.68 ± 7.51	0.005
	Did not complete college	162	12.33 ± 3.13		26.85 ± 6.67		44.48 ± 6.80	
	Completed secondary	99	12.48 ± 3.16		26.42 ± 6.77		45.66 ± 8.82	
	Elementary	40	12.80 ± 2.92		28.02 ± 8.03		49.30 ± 7.68	
**Nationality**								
	Saudi	205	12.43 ± 3.69	0.001	27.92 ± 7.11	0.195	45.53 ± 7.79	0.944
	Non-Saudi	219	13.48 ± 2.85		27.03 ± 6.91		45.58 ± 7.60	
**Occupation**								
	Blue- collar occupation	278	12.49 ± 3.14	0.001	26.93 ± 7.01	0.031	45.28 ± 7.62	0.313
	White- collar occupation	146	13.90 ± 3.46		28.47 ± 6.94		46.08 ± 7.81	

Table 4 shows the results of a multiple linear regression of factors affecting the health literacy and SMS scores. Completing college and being non-Saudi had a positively significant effect on health literacy (*p* < 0.01). On the other hand, no factors had an effect on the SMS scores (*p*˃0.05). Multiple regression was not conducted with PI as no significant relationship was noted.

**Table 4:** Factors affecting health literacy and self-medication scale scores

		BRIEF			SMS		CI		PI	
	B	t	Sig.	B	t	Sig.		B	t	Sig.
Age	0.026	2.011	0.045							
Completed college	**2.040**	3.461	0.001	**2.424**	1.881	0.061	NA			
Did not complete college	**Ref**			**Ref**						
Completed secondary	0.847	1.887	0.060	-0.449	-0.503	0.615				
Elementary	0.577	0.965	0.335	1.328	1.016	0.310				
Saudi	**Ref**					**NA**				
Non-Saudi	**1.194**	3.490	0.001							
Blue-collar occupation	**Ref**			**Ref**						
White-collar occupation	**0.010**	0.018	0.985	**-0.420**	-0.355	0.723				

Regression Weights: (Group number 1 - Default model)						
			Estimate	S.E.	C.*R*.	*P*
HL	<---	PI	− 0.026	0.024	-1.120	0.263
SMB	<---	PI	− 0.005	0.027	− 0.191	0.849
SMB	<---	HL	0.855	0.160	5.336	***

BRIEF adjusted R square = 0.085, F = 7.541, *p*-value < 0.001

SMS adjusted R square = 0.012, F = 2.310, *p*-value 0.057

**Fig. 1:**
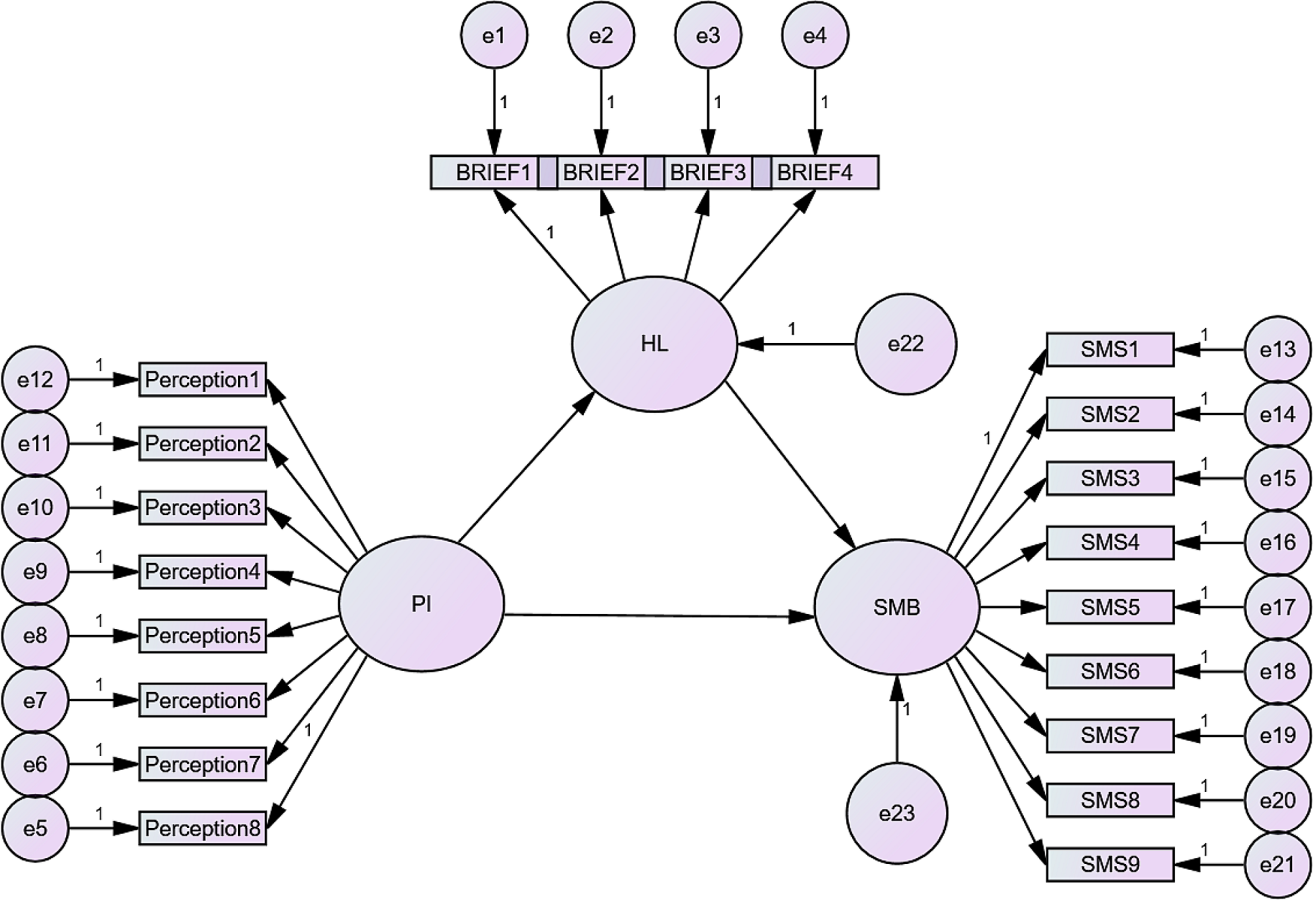
Emerging model of the influence of perception of illness and health literacy on self-medication behaviour Abbreviations: PI, perception of illness; HL, health literacy; SMB, self-medication behaviour; BRIEF, health literacy scale; SMS, self-medication scale

The SEM in Fig. 1 illustrates that PI has no effect on health literacy or SM behaviour (*p* = 0.263 and 0.84, respectively). By contrast, there was an effect of health literacy on SM behaviour (*p*<0.001).

## Discussion

This study aimed to investigate health literacy as a mediator between PI and SM among outpatients in KSA. Participants had a good PI, marginal health literacy, and propensity to SM, which means that despite their positive outlook on illness, they had a minimal level of health literacy and a tendency to rely on their own remedies. According to the findings of a number of investigations, general health state, perceived mental and physical health, and level of health literacy are the most important factors in determining whether or not an individual will self-medicate [28, 29]. For instance, demographics such as age, financial status, smoking habits, and mental health [28] and the vulnerable groups, which include the elderly, children, and patients with disabilities [29], can affect a person’s decision for self-medication. These current findings show how important it is for healthcare professionals to gauge their patients’ health literacy skills and offer proper education and counseling regarding medication use. Patient decision-making about their health can be aided by this, and the danger of unfavorable effects from self-medication can be reduced.

The results of the present study reveal a significant relationship between age and BRIEF scores, which means that an age-related difference in health literacy exists. In fact, a sizable section of the populace has been discovered to have insufficient health literacy [30]. Accordingly, those risk factors for low HL included being older, having less education and money, having been married in the past, and using health services moderately [31], and when compared, younger generations had significantly higher health literacy levels than the elder generations [32]. Also, a systematic review and meta-analysis on aging and functional health literacy found that older age was strongly associated with limited health literacy when assessed as reading comprehension, reasoning, and numeracy skills [33]. Moreover, younger participants exhibited considerably greater levels of health literacy than older participants. Such findings from these studies suggest that tailored educational programs targeting individuals with lower educational levels may be beneficial in enhancing health literacy levels and promoting better health outcomes [34, 35]. To develop targeted interventions to enhance health literacy among various age groups, it is imperative to comprehend the link between age and health literacy. It is possible to enhance people’s health outcomes by addressing age-related disparities in health literacy and assisting them in making educated decisions about their health and using the healthcare system. Executing health literacy activities and raising awareness campaigns at all educational levels and ages in various community contexts is essential. Conversely, there was no significant relationship between age, resistance to SM, and PI scores, which means that, regardless of the age of the individuals, resistance to SM and the PI remained consistent throughout the study. This result is supported by other studies. For instance, Delshad and colleagues found that age did not significantly correlate with self-medication use [36]. Conversely, self-medication habits can range among age groups, with the elderly being highly likely to self-medicate. This issue can be resolved with the use of mass media to educate the public about the risks associated with self-medication [37]. While there is no significant relationship between age, SM, and PI, the need to develop and spread public awareness campaigns regarding the risks of self-medication and the significance of safe medication practices is of importance. The demands of various age groups should be taken into consideration when designing such advertisements.

Marital status was not related to any of the scores on the health literacy (BRIEF) scale, resistance to SM, or PI scores, which supports the idea that being married or single is not predictive of health literacy levels, resistance to SM, or PI. Accordingly, although there was no correlation found between marital status and resistance to self-medication or a perception of illness, marital status was substantially linked with health literacy levels [38]. Also, another study found that age, marital status, occupation, and other socioeconomic and demographic factors of the patients, as well as their self-evaluation of their health, did not significantly correlate with health literacy [39]. The findings suggest that the participants, regardless of their marital status, are more likely to engage in healthy behaviors such as eating a balanced diet, paying attention to their health, going to the doctor regularly, and consistently putting their recommendations into practice [40]. Of note, the level of education was significantly related to all variables, which means that the participants’ level of education influences their outlook on sickness, ability to understand health information, and propensity to treat themselves with medication. Participants’ level of formal education is a powerful predictor of their ability to comprehend and act upon health information [41]. People with low levels of education find it difficult to comprehend and use health information due to low health literacy [42], which makes it difficult for them to explain their needs to healthcare practitioners.

Higher-educated people are far less likely to self-medicate, especially those with a bachelor’s or master’s degree [43, 44]. Similar to the effects of aging, health literacy can be enhanced through a combination of schooling, training, experience, and the hard-won wisdom that comes from living. Moreover, participants with higher health literacy in a Turkish study were more likely to have completed secondary school and have a positive outlook on their own health [45]. Additional research in the KSA and elsewhere predicted similar results. For example, one of the studies conducted in Saudi Arabia revealed that raising the health literacy level of a population may lead to a reduction in inappropriate self-medication [46]. One study in Egypt indicated that antibiotic resistance was strongly correlated with health literacy, even without being confounded by other factors [47]. In Jordan, a cross-sectional outpatient study revealed a statistical association between general health literacy and self-medication practice [48]. These findings show that building up the health literacy level can be one of the important factors that can reduce inappropriate self-medication.

There was a significant relationship between nationality and BRIEF scores, which means that the disparity in viewpoints towards health literacy can be attributed, in part, to linguistic as well as ethnic variables.The linguistic and ethnic nature of the gaps is seen in the trend that evidence shows that Hispanic and non-Hispanic black people generally have low levels of health literacy when compared with non-Hispanic white people [49, 50]. This may be due to language problems and a lack of knowledge of the English language, which can lead to a failure to grasp the full information on health and accessing services [51]. Therefore, in order to foster health literacy among the diversified cultures and language groups, these cultural and linguistic variations need to be considered. Such initiatives could include creating- language- and culturally sensitive health materials and treatments and endorsing the use of interpreters and multicultural staff in medical settings.

There is a significant impact of occupation on health literacy scores; this leads to self-prescription of medications and health literacy outcomes. Employment, in particular in specific fields, is shown to determine the health literacy levels of individuals according to some factors [52, 53]. For instance, one study has demonstrated that investing in healthier literacy in occupational life is cost-effective and achieves better mental and physical work capacity. This is because it improves skills and results in fewer workplace accidents; hence, promoting the maintenance of good health and work-related health literacy empowers workers to transform working conditions by themselves, and they are more likely to implement preventive measures [52]. Consequently, examining occupational settings is vital when tackling health literacy discrepancies and facilitating better health among different groups. Whereas occupation might be one of the elements affecting the levels of health literacy of a person, it is important to recognize that it is not the only decisive element. Another factor, specifically education, socioeconomic status, and cultural background, also has an influence on improving medication management and health knowledge. Also, the concept that literacy improvement at work is equal to the acquisition of skills and a better health outcome is a too simplified interpretation of reality [54]. To this end, it should not be solely occupation that is attributed to the existing disparities in health literacy, and interventions should account for the multiplex nature of the problem for it to be addressed.

There is no significant relationship between SMS scores, nationality, and source of information items, which means that the SMS outcomes remain the same regardless of the respondents’ nationalities or information sources used. SM, as argued by Agarwal et al., is a universal practice that affects people of all backgrounds and cultures equally. Nonetheless, SM is reported to be significantly more common among certain ethnic groups in the United Arab Emirates, and different cultural understandings of an illness may play a role in this discrepancy [55]. Moreover, this present study finding is in line with the results of a study conducted in the Philippines, which found that participants’ familiarity with SM had no bearing on the credibility of their informational sources [56]. Also, earlier studies have indicated that individual sectors, such as health professionals, mass media, and social media, are the main elements that determine health information and advice on self-medication and health outcomes [19, 48]. Further, it was found that there is a close connection between literacy and medication comprehension, and therefore, the ones who have higher literacy scores also report a lot of understanding of the labeled package inserts [57]. Therefore, having higher health literacy levels and supplying information that is not difficult to access can be very important to some. They can take their medicines in the correct way, and this will have a direct effect on people staying healthier. This problem underlines the value of proper communication and also of the approaches to education that will contribute to the increase of health literacy in society and thus let everybody make the correct choices about taking self-medication on their own.

Completing college and being non-Saudi had a positively significant effect on health literacy. Comparable conclusions were predicted from further research in KSA and elsewhere [30, 46], demonstrating that health literacy can be improved through a combination of formal education, receiving the right training, and experience in one’s chosen field. One of the strongest indicators of patients’ ability to understand and use health information is their level of formal education [41]. Poorly educated individuals struggle to grasp and evaluate health information, resulting in low health literacy [42]; hence, they are unable to express their needs to healthcare professionals. Concerning nationality, despite the fact that they share a cultural environment, people vary in multiple aspects, such as their theological and philosophical perspectives and the ways in which they approach life. The results of a Saudi study suggested that locals there would have difficulty using English-language health resources [58]. The use of technical phrases and medical jargon can be a significant barrier to understanding.

Studies show that nearly half of Saudis are not well-literate in health concerns. In particular, a cross-sectional survey carried out in Saudi Arabia discovered that 34.4% of participants had basic health literacy and 43.8% had intermediate health literacy [59]. It is important to point out that citizens have a far lower risk, according to the statistics, of having low health literacy [30]. Patients’ ability to read and comprehend their medical records, recommendations, and prescriptions is crucial to the functioning of any healthcare system. Health literacy standards can be implemented by public healthcare personnel to help make health information more accessible and understandable to patients. On the other hand, no factors had an effect on the SMS scores, which means that these scores were constant across a wide range of demographic considerations. SM is considered a worldwide phenomenon that occurs regardless of a person’s ethnic or socioeconomic upbringing, according to a study conducted in India [60]. Public caregivers can play a key role in reducing harmful drug use patterns by raising awareness of them, providing financial resources, and keeping tabs on how and where drugs are distributed in the community.

The SEM illustrates that PI has no effect on health literacy or SM behaviour, which means that whether participants were health-literate or self-medicated, their PI was constant. This result conforms to the findings of a study conducted in Iran that found there is no correlation between levels of health literacy and the overall mean score of PI [38]. Patients’ PI and the medication they consume are affected by the information they receive [57], and there may be a causal link between health literacy and PI if the former improves recognition and understanding of the latter. In light of these findings, it is clear that PHC personnel need to be well-versed in not only adverse events, pharmaceutical interactions, and the need to adhere to a therapeutic regimen, but also in developing and implementing educational health programmes on safe SM.

By contrast, there was an effect of health literacy on SM behaviour, which means that participants with higher health literacy are better able to weigh the benefits and drawbacks of potential medical interventions, allowing them to make more informed decisions about SM. Health literacy improves a wide range of cognitive abilities [61], including those necessary for the effective and efficient incorporation of health information, guidance, understanding, problem-solving, and judgment [62]. Poor health literacy may explain why SM has been held a culprit in previous studies [19]. As a consequence, there is an urgent requirement to reduce or eliminate SM because its impacts have further ramifications. The findings of this study supported the conclusions drawn from other lines of inquiry. For instance research conducted in Taiwan indicates a connection, between the health knowledge of teenagers and their substance use habits. This implies that possessing an understanding of health could potentially influence adolescents to refrain from engaging in behaviors, like substance abuse [63]. Given that most human actions are impacted by ideas, ideologies, and traditions, depending on the cultural and social milieu of civilisations, SM practices can evolve in different countries in varying patterns [58]. People are less likely to self-medicate incorrectly if they have a better understanding of health and wellness. Public healthcare personnel should aid the community in accessing health information, using healthcare services, and obtaining optimal health outcomes.

### Implication to primary Healthcare nursing

For primary healthcare nurses, knowing the elements that influence behaviour change is a crucial component of engaging in healthy behaviour given the rise in SM in the general population. Therefore, it is crucial to provide reports to decision-makers in the health field and policymakers in order to increase SM literacy and knowledge among all adult populations. This study contributes to the development of comprehensive and systematic initiatives of public health to raise awareness of the risks associated with SM across all demographics. Possible solutions include public health initiatives to educate the public about the risks of SM and encourage healthier lifestyles. Improving public health literacy, in conjunction with the control of other conditions, is one of the most effective ways to reduce the prevalence of SM and related risks. This is made possible by the use of resources provided by cultural, educational, and media organisations. The function of self-medication in healthcare should be explored more by nurses in the primary healthcare setting. Health policy should prioritise making sure that everyone has appropriate access to healthcare and that the general public is informed about the risks associated with self-medication through health teachings made by primary healthcare nurses.

### Study limitations

The findings of this study might not apply to the entire Saudi Arabian populace because they only represent the portion of people who can read and understand English. Further, because those with lower levels of English proficiency may be more likely to have lower levels of health literacy and participate in more self-medication behavior, the study results might underestimate the prevalence of health literacy and self-medication behavior in the Saudi Arabian population. The study may have recruited a bilingual interpreter to help with data collection from participants who were unable to comprehend or read English to address this issue. Additionally, the study might have conducted a sensitivity analysis to investigate the impact of excluding people who were not able to read.

## Conclusion

The participants had a good perception of illness, marginal health literacy and the propensity to self-medicate. The findings of this study showed that there was a significant relationship between age and BRIEF. On the other hand, there was no significant relationship between age and resistance to SM and PI scores. Marital status was not related to any health literacy (BRIEF), resistance to SM, or PI scores. Moreover, the level of education was significantly related to all variables. There was a significant relationship between nationality and BRIEF scores and between BRIEF and SMS scores. Completing college and being non-Saudi had a positive significant effect on health literacy. In contrast, no factors had an effect on the SMS scores. The SEM illustrates that PI has no effect on health literacy or SM behaviour. By contrast, health literacy has an effect on SMS behaviour. Promoting public health literacy alongside the management of other illnesses is one of the most efficient strategies for public health practitioners to reduce the prevalence of SM and the hazards associated with it.

## Data Availability

Due to the need to protect the privacy of study participants, the datasets created and/or analysed during the current investigation are not publicly accessible, but they are available upon reasonable request from the corresponding author (E. Pasay-an).
